# Predicting and Explaining Parenting Stress 3 Months After Birth Using Generalized Additive Model

**DOI:** 10.1097/CIN.0000000000001393

**Published:** 2025-12-29

**Authors:** Hannakaisa Niela-Vilen, Ilkka Suuronen, Katja Tervahartiala, Jetro J. Tuulari, Antti Airola, Riikka Korja, Linnea Karlsson, Hasse Karlsson

**Affiliations:** a Department of Nursing Science, University of Turku; b FinnBrain Birth Cohort Study, Turku Brain and Mind Centre, Department of Clinical Medicine, University of Turku; c Department of Psychiatry, Turku University Hospital and University of Turku; d Centre for Population Health Research, Turku University Hospital and University of Turku, Finland; e Department of Psychology and Speech-Language Pathology, University of Turku; f Department of Psychology, University of Jyväskylä; g The Centre of Excellence for Learning Dynamics and Intervention Research (InterLearn), University of Turku, Finland; h Turku Collegium for Science, Medicine and Technology, University of Turku, Finland; i Department of Computing, University of Turku, Turku, Finland; j Department of Clinical Medicine, Unit of Public Health, University of Turku; k Department of Child Psychiatry, Turku University Hospital, Finland.

**Keywords:** machine learning, midwifery, nursing, nursing informatics, parenting stress, postpartum period, pregnant women, stress, psychological

## Abstract

The aim of this study was to predict and explain parenting stress at 3 months after the delivery with pregnancy-related features collected in different stages of pregnancy using generalized additive modelling. Pregnant women (n=1138) were recruited for this study. The participants completed multiple questionnaires at gestational weeks 14, 24, 34, and postnatally. The Swedish Parenthood Stress Questionnaire, completed at 3 months postpartum, served as the primary outcome measure. All pregnancy-measured variables were employed as explanatory factors, and a machine learning model underwent training and testing. Initially, the model incorporated variables from gestational week 14, wherein factors associated with the mother’s personal well-being—such as quality of life and depressive symptoms—predicted parenting stress. Subsequently, the model expanded to include variables from gestational weeks 14 and 24, and based on that, also factors related to the relationship, such as avoidance and anxiety in close relationships and satisfaction in the relationship, predicted postpartum parenting stress. As the model evolved further, encompassing variables from gestational weeks 14, 24, and 34, it revealed that factors pertaining to both relationship satisfaction and the mother’s well-being continued to predict parenting stress. The study underscores the significance of addressing both maternal well-being and relationship issues during maternity care.

Pregnancy, birth, and postpartum period are times of major transitions in life and therefore have an inevitable impact on parents. Parenting stress of some degree is experienced by each parent, but sometimes stress can be detrimental to well-being.^1^ Parenting stress is defined as the experience of distress connected with parenting.^2^ It is related to the role of being a parent when parenting demands exceed the parenting resources. Thus, parenting stress is an independent phenomenon among stress conditions.^3^ Parenting stress is reportedly influenced, for example, by inadequate social support, poor health care experiences, unrealistic expectations, occupation, incomes, and health-related concerns.^4^ Research in specific patient groups, such as parents of infants in neonatal care, has reported that younger age and lower levels of education among mothers are associated with elevated stress levels.^5^ Further, some infant-related issues, for example, excessive crying, sleep problems, or health concerns, are linked to increased parenting stress.^6–8^

The phenomenon of parenting stress has been investigated since the 1970s, and, based on substantial research, it is seen as an important factor in the well-being of both parents and their children.^3^ Severe parenting stress can negatively impact the health and well-being of both mothers and their children. It may endanger the parent-infant relationship as parenting stress is related to maternal sensitivity,^9^ as well as mother-infant bonding.^10^ Parenting stress is associated with parental depression,^11^ which further threatens parent-infant interaction.^12^ Moreover, parenting stress is negatively associated with infant growth and cognitive and motor development as well as infant sleep.^5,13^ Therefore, it is essential to identify and support parents experiencing severe stress. Health care professionals—especially nurses and midwives—are in a key position to do so, as they regularly interact with pregnant and postpartum families.

The evidence on effective interventions to reduce parenting stress is inconsistent, as a “one-size-fits-all” approach is not feasible.^14^ The ability to cope with parenting stress is individual, based on personal resources as well as available social and professional support.^4,5^ Furthermore, interventions addressing parenting stress focus on different aspects, and outcome measures vary.^15^ Instead of aiming to reduce already existing parenting stress, we should try to prevent it by supporting pregnant parents early and individually in nursing and midwifery care. Thus, more understanding is needed of the sources and the primary causes of parenting stress. By identifying the pregnant parents in high risk for parenting stress, the preventive support could be better targeted and tailored.

Currently, identifying and measuring parenting stress is based on self-report scales. Reviews by Øygarden et al^16^ and Holly et al^3^ found several instruments to measure parenting stress. Many instruments demonstrated the ability to measure parenting stress both validly and reliably; however, not all of them have been validated. Most of the instruments were targeted to the postpartum period and, in particular, to mothers.^16^ However, research on the predictive factors of parenting stress remains limited.^17^ Longitudinal study by Hames et al^5^ aimed to discern the sources of parenting stress for the purpose of forecasting stress levels following the hospitalization period of infants. The results showed that the perception of a more severe baby’s illness predicted more parenting stress at hospital discharge. Increased symptoms of antenatal depression and anxiety seem to be associated with elevated parenting stress during the first year after the birth.^18,19^ Moreover, maternal prenatal attachment style seems to predict postnatal parenting stress.^17,20^ Therefore, further research is needed to better understand the factors contributing to severe parenting stress to provide nurses and midwives with more knowledge and tools to support parents effectively.

## AIMS

The aim of this study was to predict and explain parenting stress at 3 months after the delivery with pregnancy-related features collected in different stages of pregnancy using generalized additive modelling. This was an exploratory study geared to leverage machine learning algorithms for predicting parenting stress, with the aim of identifying innovative avenues for healthcare interventions and nursing informatics, thereby enhancing overall family well-being.

## METHODS

### Design and Data Collection

The data are part of the prospective observational FinnBrain Birth Cohort Study in which the effects of prenatal and early life stress exposure on child health and brain development were investigated. The participating women (n=3808) were recruited at gestational week 12 in Southwest Finland from 2011 to 2015. The inclusion criteria for participation were sufficient knowledge of Finnish or Swedish and a normal ultrasound screening result. During pregnancy, the participating women completed questionnaires at gestational weeks 14, 24, and 34. Postnatally, the cohort participants were followed using questionnaires at different intervals.^21^ Regarding this study, the questionnaires completed during pregnancy and at 3 months postpartum were used.

### Participants

From the original data set, a total of 1196 participants who had completed the main outcome measure at 3 months postpartum were originally included in this study through convenience sampling. After the data cleaning and preprocessing, the final sample consisted of 1138 participants.

### Measures

The Swedish Parenthood Stress Questionnaire (SPSQ) completed at 3 months postpartum was used as the main outcome measure. The SPSQ is a modified version of the Parent Domain from the Parenting Stress Index, consisting of 34 items. These items assess general caregiving experiences, feelings of parental incompetence, perceptions of parenthood as challenging, limitations on personal interests due to parental responsibilities, social interactions, family-related social experiences, and the parent’s physical health. The mothers assessed on a 5-point Likert scale the degree to which they agreed or disagreed with each item. Each item is scored from 1 to 5; thus, the total score of the questionnaire varies between 34 and 170 and describes the General Parenting Stress. A higher total score indicates higher parenting stress. The items can also be divided into 5 subscales: incompetence, role restriction, social isolation, spouse relationship problems, and health problems. In this study, only the General Parenting Stress, that is, the total mean score was used.^2,22^

All the variables measured during pregnancy at the 3 measurement points in the FinnBrain Cohort Study were used as explanatory factors. The measures and instruments are shown in Table 1. Background information variables included family characteristics, marital status, living environment, education, income, profession, family diseases, satisfaction with physical and mental health, satisfaction with income, marital satisfaction and the use of medication, origins, as well as native language. Further, internationally validated instruments were used to measure quality of life, maternal-foetal attachment, daily hassles, resilience, sleep, dental anxiety, depressive symptoms, substance use, trauma and distress, anxiety, close relationships, bonding, sense of coherence, and parental reflexive functioning.^21,23–40^

**Table 1:** Measures Used in the Models

	Measurement Points		
	T1 Gestational Week 14	T2 Gestational Week 24	T3 Gestational Week 34
Background information, family characteristics, socioeconomic status^21^	X		
WHO Quality of Life Scale (WHOQOL)^23^	X		X
Maternal Fetal Attachment Scale (MFAS)^24^	X	X	X
Modified Daily Hassles Scale^25^	X	X	X
The Connor-Davidson Resilience Scale (CD-RISK)^26^	X		
Prenatal Parental Reflective Functioning P-PRFQ^27^			X
Basic Nordic Sleeping Questionnaire (BNSQ)^28^	X	X	X
Athens Insomnia Scale (AIS)^29^	X	X	X
Symptom Checklist-90, SCL-90^30^	X	X	X
Modified Dental Anxiety Scale (MDAS)^31^	X		X
The Anxiety Symptom Scale (ASS)^32^			X
Depression Scale, EPDS^33^	X	X	X
Substance use during pregnancy^21^	X		X
Trauma and Distress Scale (TADS)^34^	X		
Pregnancy Related Anxiety Questionnaire PRAQ^35^	X	X	X
Experiences in Close Relationship Scale (ECR-R)^36^		X	
Parental Bonding Instrument PBI^37^		X	
The Sence of Coherence (SOC)^38^		X	
Oral Health-Related Quality of Life (OHIP)^39^			X
Revised Dyadic Adjustment Scale, RDAS^40^			X

### Procedures and Data Analysis

In this study, we wanted to (1) investigate the ability of a machine learning model to predict the severity of parenting stress, (2) identify factors associated with parenting stress, and (3) observe the change in prediction accuracy as information collected at each trimester of pregnancy is being incrementally introduced into the model. To this end, we trained and tested an interpretable machine learning regression model with tabular questionnaire data collected from the participants at gestational weeks 14, 24, and 34 (henceforth referred to as time points T1, T2, and T3) with a measure of parenting stress as quantified by the total mean SPSQ score as the outcome variable using a repeated cross-validation strategy. This allowed us to evaluate the model’s predictive performance at each time point and observe how accuracy improved as more information became available. In addition, to investigate the factors associated with parenting stress at each time point, we retrained the model with data from all participants with all variables available at that time point and ranked the predictors by their importance for making predictions.

### Data Preprocessing

The tabular data originally consisted of 1196 rows (participants) and 3039 columns (variables), including both numerical and categorical variables. Nine of the rows were found to be duplicates and were removed. In addition, the outcome variable value was missing for 49 participants, and the corresponding rows were removed. Only the variables collected at the time points of interest, T1 (gestational week 14), T2 (gestational week 24), and T3 (gestational week 34) were kept, as well as the total SPSQ mean value at 3 months postpartum, which was used as the outcome variable, while the rest of the columns were discarded. For removing columns with a large number of missing values, we adopted a conservative strategy, setting the minimum condition for keeping a column at 20% of values not missing. This data cleaning procedure resulted in 3 data sets of 1138 rows and 262, 334, and 492 columns, for time points T1, T2, and T3, respectively, with each consecutive time point-specific data set also including the variables from the preceding time points.

### Analysis Pipeline Definition

Further preprocessing steps included imputation of missing values and standardizing the numerical values by removing the mean and scaling to unit variance. In case of missing categorical values, a constant value indicating a missing value was used, whereas missing numerical values were imputed with the mean value of the most similar datapoints using the k-nearest neighbors (KNN) imputation method.^41^ These preprocessing steps were implemented as a part of the machine learning pipeline to avoid any information leakage from the test data to the model that might occur due to performing such operations before splitting the data.

### Machine Learning Model

For predicting the outcome variable, we used an explainable boosting machine (EBM)^42^ regression. EBM is a machine learning model based on a generalized additive model (GAM) and, as such, is highly interpretable as the contribution of each predictor term on the predicted outcome can be independently observed. EBM additionally includes a small number of automatically detected pairwise interaction terms, making its predictive power comparable to less interpretable full-complexity models (eg, random forests).^43^ The combination of high predictive power and interpretability made EBM the model of choice for the present task.

To evaluate the machine learning model’s accuracy in predicting the outcome variable, we used 10 times repeated 5-fold cross-validation strategy. In brief, k-fold cross-validation is a conventional technique for evaluating a machine learning model by partitioning the data set into k distinct subsets (aka. folds), each of which is in turn used for testing the model while the rest of the data (k-1 folds) are used for training the model. This results in k test scores, which can then be averaged for a robust estimate of model test performance. In repeated k-fold cross-validation, the procedure is repeated with different random partitions for a more accurate estimate of model performance. Model performance was measured in terms of *R* ^2^, representing the proportion of variance in the outcome variable explained by the predictors.

**Figure 1:**
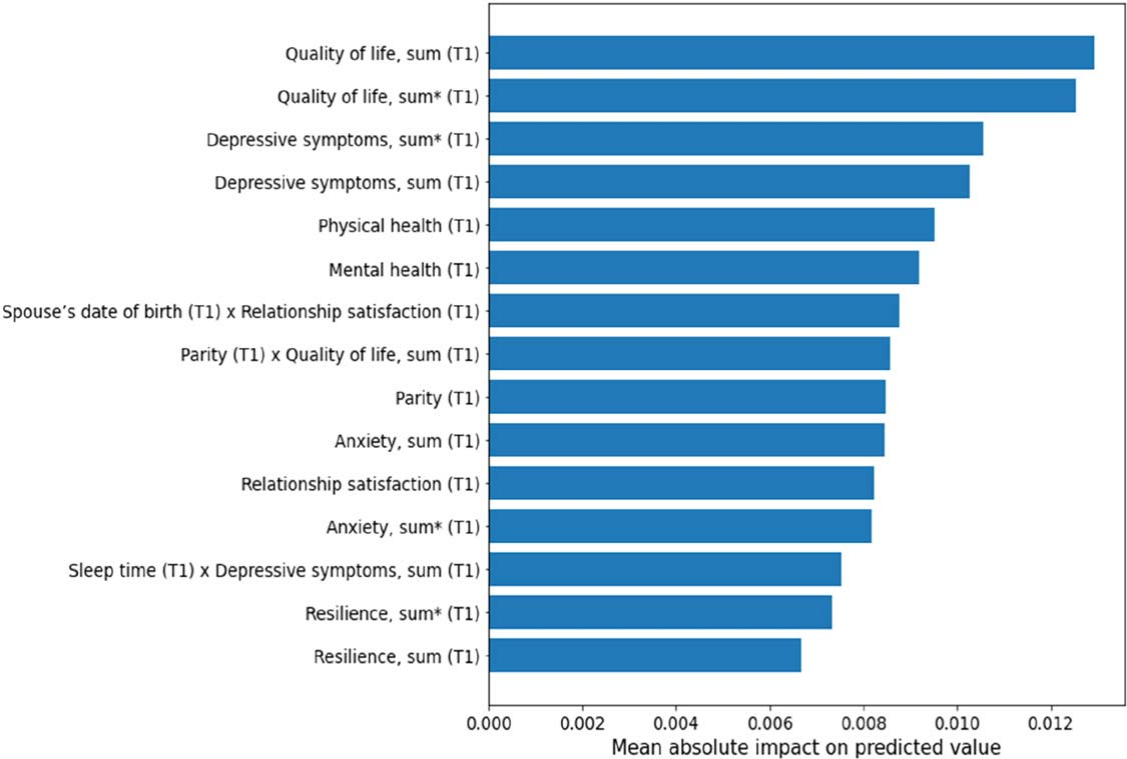
Significant predicting factors at pregnancy time point 1 (T1). Predictors and interactions are ranked based on the mean absolute contribution of the corresponding additive term to the final predicted value over the data set. “Sum” refers to the total score of the items in an instrument, where possible missing information is replaced with the mean of other answers; “sum*” refers to a direct sum of the scale without replacing missing information.

**Figure 2:**
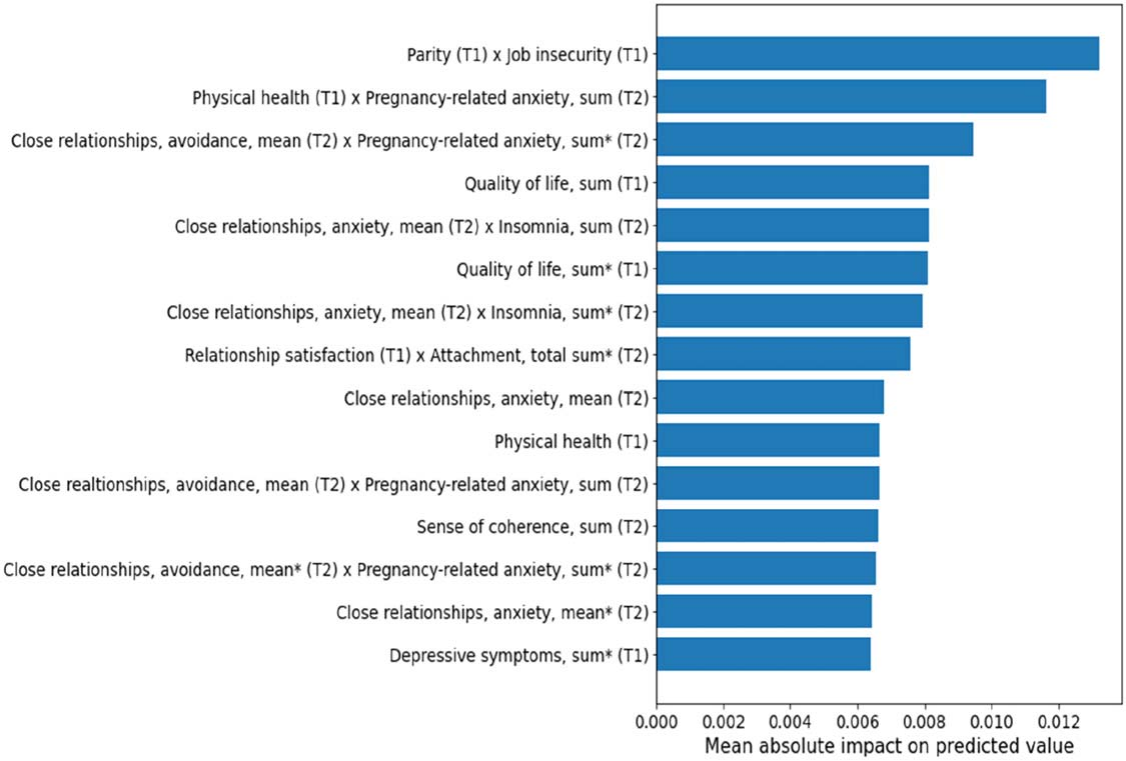
Significant predicting factors at pregnancy time points 1 (T1) and 2 (T2). Predictors and interactions are ranked based on the mean absolute contribution of the corresponding additive term to the final predicted value over the data set. “Sum” refers to the total score of the items in an instrument, where possible missing information is replaced with the mean of other answers; “sum*” refers to a direct sum of the scale without replacing missing information.

**Figure 3:**
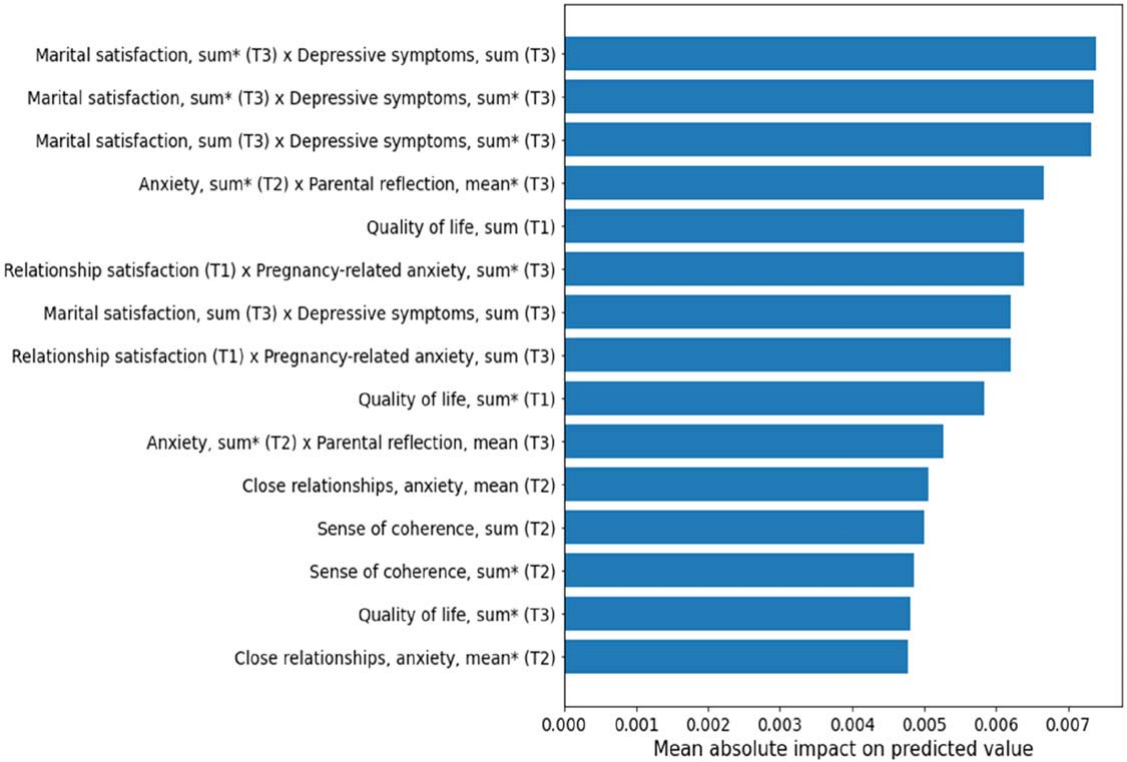
Significant predicting factors at pregnancy time points 1 (T1), 2 (T2), and 3 (T3). Predictors and interactions are ranked based on the mean absolute contribution of the corresponding additive term to the final predicted value over the data set. “Sum” refers to the total score of the items in an instrument, where possible missing information is replaced with the mean of other answers; “sum*” refers to a direct sum of the scale without replacing missing information.

While cross-validation estimates machine learning model performance reliably, it does not result in a single, unambiguous trained model to investigate further. For the purpose of feature importance analysis, we trained a final model at each time point using the data from all participants with variables collected up to that time point, and used the model to rank the predictors and potential pairwise interactions based on their mean absolute contribution to the mean predicted outcome over the dataset and visualized the highest-ranking predictors and interactions in horizontal bar plots (Figures 1–3). In this way, we were able to investigate which predictors were most important for determining the predicted outcome at each time point. Although this approach results in an intuitive basis for ranking the predictors based on their importance, it should be noted that the direction of the association is not available for such a global explanation of the model.

The machine learning analysis was implemented in Python using Scikit-learn^44^ (version 1.2.1) machine learning framework and interpret.ml^42^ (version 0.3.2). The default hyperparameter values were used for the predictive EBM model and the preprocessing steps of the analysis pipeline, with the exception of KNN imputation, for which the neighbourhood size was set at 10 (from the default 5) for a more robust imputation neighborhood.

### Ethical Considerations

The Ethics Committee of the Hospital District of Southwest Finland (currently The Ethics Committee of the Wellbeing Services County of Southwest Finland) has approved the FinnBrain Birth Cohort Study protocol on June 14, 2011, with the number ETMK: 57/180/2011. A written informed consent was obtained from all parents participating in the study.

## RESULTS

The median age of participating mothers (n=1138) was 31.0 years, ranging from 19.0 to 44.0 years. Of these mothers, 53% were primiparous, 43% were multiparous, and information was missing for the remaining 4%. The majority (93.1%) were married or cohabiting with a partner. Educational levels were generally high, with 69.2% having attained a university-level education. The infants of the participating mothers were born at a median gestational week of 40 (range=25.9 to 42.4).

When considering the model trained on the variables only from T1 (gestational week 14), the factors predicting parenting stress were related to the mother’s own well-being, such as quality of life, depressive symptoms, and the participants’ satisfaction with her own physical and mental health (Figure 1). The model trained only on T1 variables achieved a cross-validated mean test performance of *R* ^2^=0.19.

When the variables from both T1 and T2 (gestational weeks 14 and 24) were included, in addition to the factors related to mother’s own well-being (eg, quality of life, satisfaction with her own physical health, insomnia, and pregnancy-related anxiety) the factors related to the relationship, such as avoidance and anxiety in close relationships and satisfaction in relationship, predicted postpartum parenting stress (Figure 2). The model trained on the variables of the first 2 time points achieved a cross-validated mean test performance of *R* ^2^=0.25.

In the last model, all the variables from T1 to T3 (gestational weeks 14, 24, and 34) were included. Both factors related to the relationship satisfaction and factors related to the mother’s own well-being predicted parenting stress (Figure 3). The model trained on the variables of all 3 time points achieved a cross-validated mean test performance of *R* ^2^=0.29.

The cross-validated results of our analysis indicate reasonable mean test performance across all time points, with significant improvements as new information is introduced to the model at each stage (from mean *R* ^2^=0.19 at T1 to *R* ^2^=0.29 at T3).

## DISCUSSION

Based on the machine learning models used in this study, maternal well-being and relationship satisfaction during pregnancy appeared to predict parenting stress at 3 months postpartum. This innovative approach allowed us to describe the markedly multifaceted underpinnings of parenting stress. Predicting postpartum parenting stress during pregnancy presents a novel opportunity to enhance the health and well-being of both the mother and her child. The findings of this study support a shift from reactive to preventive care, which is an important aspect of nursing and midwifery practice.

In this study, maternal well-being during pregnancy was broadly considered, encompassing subjective evaluations of depressive and anxiety symptoms, sleep quality, and quality of life, elements often already integrated into nursing and midwifery care. The machine learning models used revealed a link between antenatal depressive and anxiety symptoms and postnatal parenting stress. Notably, depressive symptoms already in early pregnancy were associated with postpartum parenting stress, whereas earlier studies had demonstrated this association only in the third trimester.^18,19^ The results of this study suggest that early identification and intervention of antenatal depressive and anxiety symptoms might prevent or decrease parenting stress. An association between maternal depressive symptoms and parenting stress has been confirmed in numerous previous studies; however, the emphasis has typically been on postnatal depressive symptoms.^10,45^ Since screening for depressive symptoms during pregnancy is common and strongly recommended by the American College of Obstetricians and Gynecologists, such screening and early initiation of treatment could significantly contribute to the holistic well-being of a pregnant woman, including the prevention of excessive parenting stress. Nurses and midwives play a critical role in assessing maternal well-being during pregnancy, and the insights from this study could be integrated into prenatal education programs.

Quality of life (QoL) during pregnancy appeared to be a significant factor in predicting parenting stress 3 months postpartum. QoL is a multidimensional concept that assesses an individual’s overall well-being, emphasizing the biopsychosocial aspects of their welfare. Even in uncomplicated pregnancies, mothers experience many physical and emotional changes that may have an impact on QoL. A high level of parenting stress has been associated with a low level of QoL among parents of children with autism spectrum disorder.^46^ However, there are no previous studies conducted on a scale comparable to the sample size in this study. In addition to depressive symptoms and QoL, sleep is an important contributor to well-being. Although maternal sleep issues have been previously linked to parenting stress,^47^ sleep during pregnancy was not considered significant based on the machine learning analyses performed in this study.

In the Finnish maternity care context, public health nurses, who are responsible for the care of pregnant women, should be aware of the predictive nature of depressive and anxiety symptoms as well as the importance of the individual’s experience of quality of life. Depressive symptoms in pregnant women are already routinely screened during antenatal visits. In the future, we should also consider the possible benefits of including the assessments of anxiety symptoms and the quality of life, especially if depressive symptoms are detected. Data from such nursing assessments can help tailor care plans and develop individualized support strategies for both the pregnancy and postpartum periods.

In addition to maternal well-being, factors related to her close relationships during the last trimester of pregnancy were shown to be significant when predicting postnatal parenting stress. As pregnancy progresses, the focus naturally shifts from self-related concerns to relationship-related matters. Therefore, it seems logical that relationship issues gain significance toward the end of pregnancy. However, parents are not always prepared for the changes in their relationships during the perinatal period.^48^ High-quality interparental support reduces stress levels during pregnancy and, by extension, helps prevent problems with parent-infant bonding. A large Norwegian study showed that maternal relationship satisfaction slightly increased during pregnancy but decreased around childbirth.^49^ Nurses, midwives and all other health care professionals caring for pregnant women need to have knowledge and skills about how to broach relationship issues at maternity clinic visits. Satisfaction with a relationship is a sensitive matter that depends on individual factors.^50^ Therefore, a thorough examination of this issue by health care professionals is essential. Nurses and midwives are well-positioned to identify these concerns and, by extension, to educate and counsel expectant parents on their holistic health.

### Strengths and Limitations

The earlier prediction of parenting stress allows for more extended intervention period to prevent adverse effects. However, prediction alone is insufficient without tailored and effective interventions to prevent or mitigate parenting stress. A notable strength of this study is its large sample size, encompassing a considerable number of measured variables. Although this study used convenience sampling, the sample closely reflects the original cohort.^21^ The original cohort was recruited over a decade ago, and some stressors may have evolved in recent years, for example, due to the pandemic. However, many pregnancy-related stressors are enduring in nature and continue to provide valid and relevant insights. The machine learning method employed in this study enables the utilization of multiple variables at different time points as potential predictors of postnatal parenting stress. These methods offer novel and innovative approaches to analyzing health-related big data. It is noteworthy that although the degree of explanation increased, it did not increase proportionally with the number of variables added to the model. Moreover, to utilize this machine learning model as a tool for intervention development, the accuracy of the model needs further enhancement.

Another limitation is that the global explanation for the model does not unambiguously reveal the direction of the association between the dependent and independent variables, although it is possible to study the direction of the association locally, that is, on a case-by-case basis. Further research with various methods is needed to better understand these associations. An important point to note is that this study solely focused on prenatally measured factors. It is crucial to acknowledge that postnatal factors also play a role in parenting stress. In the future, integrating both prenatal and postnatal factors would yield significant new insights. Moreover, investigating parenting stress beyond 3 months postpartum would offer further insights into this multifaceted phenomenon.

## CONCLUSION

The ability to predict parenting stress could open new possibilities to nurses and midwives in maternity care for early intervention and the prevention of excessive parenting stress. These results provide clear targets for holistic assessment and interventions, as many categories that best explain increased parenting stress levels are already included in routine healthcare screenings during pregnancy. Notably, the integration of machine learning into maternal care highlights the evolving role of nursing informatics in advancing data-driven, personalized health care. In addition, these findings inform the development of the machine learning model as a tool for creating interventions. Understanding the significance of various factors and the potential cumulative burden on individuals can offer clear indicators to nurses, midwives, and other health care professionals, enabling them to customize support for parents.
